# Insights into the structure of mature streptavidin C1 from *Streptomyces cinnamonensis* reveal the self-binding of the extension C-terminal peptide to biotin-binding sites

**DOI:** 10.1107/S2052252520015675

**Published:** 2021-01-11

**Authors:** Byeong Jun Jeon, Sulhee Kim, Min-Seok Kim, Ji-Ho Lee, Beom Seok Kim, Kwang Yeon Hwang

**Affiliations:** a Department of Plant Biotechnology, School of Life Sciences and Biotechnology for BK21 PLUS, Institute of Life Science and Natural Resources, Korea University, 145 Anam-ro, Seongbuk-gu, Seoul 02841, South Korea; b Department of Biotechnology, School of Life Sciences and Biotechnology for BK21 PLUS, Institute of Life Science and Natural Resources, Korea University, 145 Anam-ro, Seongbuk-gu, Seoul 02841, South Korea

**Keywords:** streptavidin, biotin, antifungal activity, *Streptomyces cinnamonensis*, self-binding

## Abstract

The members of the avidin protein family are well known for their high affinity towards d-biotin and their structural stability. These properties make avidins a valuable tool for various biotechnological applications. In the present study, two avidin-like biotin-binding proteins (named streptavidin C1 and C2) from *Streptomyces cinnamonensis* were newly identified while exploring antifungal proteins against *Fusarium oxysporum* f. sp. *cucumerinum*. Streptavidin C1 reveals a low correlation (a sequence identity of approximately 64%) with all known streptavidins, whereas streptavidin C2 shares a sequence identity of approximately 94% with other streptavidins. Here, the crystal structures of streptavidin C1 in the mature form and in complex with biotin at 2.1 and 2.5 Å resolution, respectively, were assessed. The overall structures present similar tetrameric features with *D*_2_ symmetry to other (strept)avidin structures. Interestingly, the long C-terminal region comprises a short α-helix (C-Lid; residues 169–179) and an extension C-terminal peptide (ECP; residues 180–191) which stretches into the biotin-binding sites of the same monomer. This ECP sequence (–^180^VTSANPPAS^188^–) is a newly defined biotin-binding site, which reduces the ability to bind to (strept)avidin family proteins. The novel streptavidin C1 could help in the development of an engineered tetrameric streptavidin with reduced biotin-binding capacity as well as other biomaterial tools.

## Abstract 2

The structural analysis of mature streptavidin C1 from *S. cinnamonensis* is reported.

## 1. Introduction   

Avidin from chicken (*Gallus gallus*) egg white is a naturally glycosylated protein, whereas streptavidin is a nonglycosyl­ated protein secreted by *Streptomyces avidinii*. These proteins have long been studied for a diverse array of medical and biotechnological applications. Avidin and bacterial strept­avidin, which have a homotetrameric quaternary structure, are known to exhibit an extremely high affinity (*K*_d_ values of approximately 10^−15^ *M*) for biotin (Green, 1990 ▸). The high-affinity avidin–biotin system is unique, and its versatile applications are primarily based on the chemistry of biotin conjugation (Diamandis & Christopoulos, 1991 ▸). These applications include protein detection, microscopy, diagnosis, drug delivery and others (Laitinen *et al.*, 2007 ▸; Lesch *et al.*, 2010 ▸). Although highly useful, the aforementioned system has certain limitations, which frequently result from the homotetrameric assembly of the four high-affinity biotin-binding sites, with the tight biotin binding making streptavidin un­suitable for the affinity purification of biotinylated molecules. It would be ideal to develop an engineered streptavidin with a reversible biotin-binding capability. However, unexpected cross-linking could occur, and precise quantification is impossible as the binding-site occupancy cannot be verified. Several studies have been carried out to minimize and alter the tetrameric assembly in order to decrease the stoichiometry of avidins (Howarth *et al.*, 2006 ▸; Laitinen *et al.*, 2006 ▸). Attempts to minimize the tetrameric configuration of avidins via rational design have been less successful (DeMonte *et al.*, 2013 ▸; Hytönen *et al.*, 2005 ▸; Laitinen *et al.*, 2006 ▸; Lee *et al.*, 2016 ▸; Nordlund, Hytönen, Hörhä *et al.*, 2005 ▸). To date, numerous proteins belonging to the avidin family have been explored, including those originating from various organisms ranging from bacteria to fungi, birds, reptiles and fish (Chaiet & Wolf, 1964 ▸; Green, 1990 ▸; Keinänen *et al.*, 1994 ▸; Määttä *et al.*, 2009 ▸; Takakura *et al.*, 2009 ▸; Taskinen *et al.*, 2013 ▸). Despite their functional similarities, streptavidin and avidin present different amino-acid sequences in their primary structures. The degree of sequence identity between these proteins is approximately 30%. Avidin has a high isoelectric point (pI = 10.5), whereas streptavidin has a low isoelectric point (pI = 6.1–7.5) compared with avidin. The high pI of avidin can cause nonspecific binding to various biological components, thus limiting its use in specific applications. On the other hand, the amino-acid sequences of streptavidin V1 and streptavidin V2 purified from *S. venezuelae* were identical to that of the original streptavidin, except for the first and ninth amino acids, respectively (Bayer *et al.*, 1995 ▸).

Within the tetrameric assembly of the avidins, three types of monomer–monomer interactions (Livnah *et al.*, 1993 ▸) exist. The 1–2 interaction involves a tryptophan residue (position 110 in egg-white avidin) that is donated from one monomer to the biotin-binding site of the adjacent monomer. The 1–3 interaction is relatively small and involves approximately 3–4 participating residues from each monomer. The 1–4 inter­action is the most important, with a contact area of 1200–1500 Å^2^ per monomer, and is also termed a sandwich-like interaction (Meir *et al.*, 2009 ▸). Recently, the discovery of high-affinity dimeric avidins from bacterial sources, including bacterial avidin-like proteins from *Rhizobium etli* (rhizavidin), *Shewanella denitrificans* (shwanavidin) and *Hoeflea phototrophica* DFL-43 (hoefavidin), has been reported, although the high affinity for biotin was maintained in these proteins (Avraham *et al.*, 2015 ▸; Helppolainen *et al.*, 2007 ▸; Meir *et al.*, 2009 ▸, 2012 ▸). Structural analyses of these proteins revealed that they maintain the unique basic features of the avidin family, in which each monomer displays a near-identical topology and quaternary structure, with similar interactions to the 1–4 interaction of the tetrameric avidins, thus forming a sandwich-like dimer. The dimeric structure lacks the 1–2 and 1–3 interactions and the critical crossover tryptophan residue that distinguish the tetrameric avidins.

To elucidate the strategies to be applied in engineering streptavidin with biotin-binding ability, it is crucial to understand the unique structural features of (strept)avidin, its biotin-binding pockets and the quaternary interfaces. In the present study, we discovered two novel avidin-like biotin-binding proteins (named streptavidins C1 and C2) from *S. cinnamonensis* while exploring antifungal proteins against *Fusarium oxysporum* f. sp. *cucumerinum*. Interestingly, strept­avidin C1 reveals a low correlation (a sequence identity of approximately 64%) with all known streptavidins, whereas streptavidin C2 shares a sequence identity of approximately 94% with other streptavidins. We determined crystal structures of streptavidin C1 (191 amino acids) in the mature form and as a complex with biotin at 2.1 and 2.5 Å resolution, respectively. The well ordered long C-terminus comprises a short α-helix and a C-terminal tail which stretches into the biotin-binding sites of the same monomer. The structures clearly revealed unique structural and compositional features in comparison with other avidin-like proteins. Moreover, we further describe the structural analysis and biochemical characterization of streptavidin C1. This novel member of the (strept)avidin family could be used in diverse biotin-based nanotechnologies or may act as a building block in the production of new bioinspired materials.

## 2. Experimental procedures   

### 2.1. Identification, production and purification of antifungal proteins   

*S. cinnamonensis* strain KPP02129 was grown on tryptic soy agar plates for three days at 28°C. A single colony on the plate was transferred to a 1 l Erlenmeyer flask containing 100 ml tryptic soy broth (TSB) at 28°C for three days on a rotary shaker at 200 rev min^−1^. After three days, modified TSB (4 g tryptone, 0.5 g soytone, 2.5 g dextrose, 5 g sodium chloride, 2.5 g dipotassium hydrogen phosphate in 1 l water) medium (1 l) was inoculated with 1%(*v*/*v*) of the preculture and incubated at 28°C on a rotary shaker at 200 rev min^−1^ for five days. The 1 l culture was centrifuged at 8000*g* for 30 min to separate the cells from the broth, and solid ammonium sulfate was then added to the culture broth to 95% saturation at 4°C. The resulting precipitate was harvested via centrifugation at 13 000*g* for 30 min at 4°C. The pellet was dissolved in approximately 20 ml 20 m*M* Tris–HCl pH 8.0 and dialyzed with the same buffer overnight three times. The resulting protein solution was loaded onto a 5 ml HiTrap Q HP column (GE Healthcare Bio-Sciences, Uppsala, Sweden) equilibrated with 20 m*M* Tris–HCl pH 8.0. After washing the column with the same buffer, the protein was eluted with a stepwise gradient of increasing NaCl concentration (100, 150, 250, 300 and 400 m*M* and 1 *M* NaCl). The eluted fractions were assayed for antifungal activity against *F. oxysporum* f. sp. *cucumerinum* (FOC) using a paper-disk assay and were then separated via SDS–PAGE on 15% gels. Proteins were stained with Coomassie Brilliant Blue G-250 or by the silver-staining method. The band containing the active fraction was excised. Protein identity analysis of the excised band was performed using a UHPLC-MS/MS system (Thermo Scientific Dionex Ultimate 3000) equipped with an analytical column (Acclaim PepMap RSLC 50 µm × 15 cm, nanoViper, C18, 2 µm, 100 Å; Thermo Scientific).

### 2.2. Primary-structure analysis and protein expression   

Avidin-like genes were amplified by PCR using the DNA of *S. cinnamonensis* strain KPP02129 as a template. The primer set used for PCR included C1F-NdeI and C1R-XhoI or C2F-NdeI and C2R-XhoI (Supplementary Table S1). 2× PreMIX-HF (Macrogen Co., Seoul, South Korea) was then added to the reaction. The amplification was performed with an initial denaturation step at 95°C for 5 min followed by 30 cycles of denaturation at 94°C for 1 min, annealing at 60°C for 1 min and extension at 72°C for 90 s, and a final extension at 72°C for 3 min. The amplified products were cloned into the pTOP TA V2 vector (Macrogen) for TA cloning and were sequenced. The resulting sequences and the avidin-related protein sequences were aligned with *ClustalW* (https://www.genome.jp/tools-bin/clustalw). *ESPript* (http://espript.ibcp.fr/ESPript/cgi-bin/ESPript.cgi) was used for alignment, visualization and manipulation. The amplified streptavidins C1 and C2 were cloned into pET-21a vector using the NdeI and XhoI restriction enzymes. Moreover, C-terminal deletion mutants of streptavidin C1 (ΔC1, amino acids 1–161, and ΔC2, amino acids 1–158) were cloned into the same vector. The recombination plasmids were transformed into *Escherichia coli* strain BL21 harboring one of the expression vectors. The cells were grown in Luria–Bertani (LB) broth containing ampicillin at 37°C until late log phase (OD_600_ = 0.6–0.8). The cultures were induced with 1 m*M* isopropyl β-d-1-thio­galactopyranoside (IPTG) and then shaken for a further 4 h at 37°C or 16 h at 18°C. The cells were harvested via centrifugation and tested for solubility.

### 2.3. Protein production and purification   

Expression of all of the recombinant proteins was induced with 1 m*M* IPTG for 16 h at 18°C in LB medium containing ampicillin. After harvesting via centrifugation at 6900*g* for 1 h, the total proteins were resuspended in a buffer consisting of 20 m*M* Tris–HCl pH 8.0, 100 m*M* NaCl, 2 m*M* β-mercapto­ethanol and disrupted by sonication. Cell debris was removed via centrifugation at 9500*g* at 4°C for 1 h. The supernatant was passed through an Ni–NTA affinity column (5 ml HisTrap HP, GE Healthcare) equilibrated with binding buffer (20 m*M* Tris–HCl pH 8.0, 100 m*M* NaCl, 2 m*M* β-mercaptoethanol) and then eluted with elution buffer (20 m*M* Tris–HCl pH 8.0, 100 m*M* NaCl, 2 m*M* β-mercapto­ethanol, 500 m*M* imidazole). Fractions were examined using SDS–PAGE and those that contained protein were pooled. The protein was further purified via anion-exchange chromatography and eluted with a linear NaCl gradient in 20 m*M* Tris–HCl pH 8.0, 2 m*M* dithiothreitol (DTT). The purified protein was concentrated using 10K Amicon ultracentrifugal filters (Millipore, Bedford, Massachusetts, USA) and further purified via size-exclusion chromatography using a Superdex 200 26/60 column (GE Healthcare, Little Chalfont, UK) with a final buffer consisting of 20 m*M* Tris–HCl pH 8.0, 100 m*M* NaCl, 2 m*M* DTT.

### 2.4. *In vitro* antifungal assay   

The streptavidin C1 and C2 proteins were assayed to evaluate their antifungal activity against FOC using a 96-well plate. Various concentrations of the proteins (1–80 µg ml^−1^) and conidia suspension (1.5–2.0 × 10^3^) were applied to each well of the 96-well plate containing 100 µl half-strength potato dextrose broth. After incubation at 28°C for 72 h, the plate was visually inspected for concentrations that inhibited fungal growth. To assess whether excess biotin would abolish the activity of streptavidins C1 and C2, d-biotin (4 µg ml^−1^) was added.

### 2.5. Thermal stability   

To determine thermal stability, the proteins (0.1 mg ml^−1^) were combined with 1× SDS sample buffer consisting of 50 m*M* Tris–HCl pH 6.8, 3% β-mercaptoethanol, 1%(*w*/*v*) SDS, 10% glycerol and were incubated for 20 min at various temperatures (25, 50, 60, 70, 75, 80, 90 and 100°C). The proteins were also incubated with or without β-mercapto­ethanol in 1× SDS sample buffer at room temperature (RT) or at 95°C in the absence and presence of biotin (1 mg ml^−1^). The reaction mixtures were then subjected to SDS–PAGE. To evaluate the transition temperature, the proteins were evaluated in a manner in which half of the proteins were tetrameric and other half were monomeric in the absence and presence of biotin. Streptavidin C1, streptavidin C2, strept­avidin and avidin were diluted with PBS containing 0.05% Tween 20 (PBS-T) to a concentration of 1 mg ml^−1^ for Tycho NT.6 experiments. The final concentration of d-biotin was 4 mg ml^−1^. The samples were then loaded as duplicates into Tycho NT.6 capillaries (NanoTemper Technologies, Munich, Germany; catalog No. TY-C001). The temperature-inflection values (*T*_i_) were obtained via automated data analysis.

### 2.6. Analysis of strepavidin–biotin interactions via microscale thermophoresis   

Microscale thermophoresis (MST) assays were performed to study the protein–ligand interaction using a Monolith NT.115 instrument (NanoTemper Technologies). Recombinant streptavidin C1-His, streptavidin C2-His, streptavidin-His (ab78833), ΔC1-His and ΔC2-His proteins were labeled using the Monolith His-Tag Labeling Kit RED-tris-NTA 2nd Generation kit according to the manufacturer’s instructions. Nonlabeled biotin at increasing concentrations (0.3052–10 000 p*M*) in PBS-T was incubated for 10 min at room temperature with 200 p*M* labeled streptavidin-His. Moreover, nonlabeled C-Lid_ECP or ECP in PBS-T buffer at increasing concentrations (2.4414–80 000 n*M*) was incubated for 10 min at room temperature with 200 n*M* labeled ΔC1-His and ΔC2-His. The amino-acid sequences of C-Lid_ECP and ECP were synthesized as –^169^SAADVEKARQLGVTSANPPASDGE^191^– and –^178^LGVTSANPPASDGE^191^–, respectively (Peptron, Daejeon, South Korea). Thereafter, the samples were loaded into capillaries (Mo-Ko22 Monolith NT.115) and were evaluated at 25°C with 40% LED power and 60% MST power. The results were processed using *MO Affinity Analysis* and *GraphPad Prism*. Each measurement was performed in triplicate at the same MST power.

### 2.7. Size-exclusion chromatography with multi-angle light scattering (SEC-MALS)   

SEC-MALS experiments were performed using a fast protein liquid-chromatography system (GE Healthcare) connected to a Wyatt MiniDAWN TREOS MALS instrument and a Wyatt Optilab rEX differential refractometer. A Superdex 200 10/300 GL (GE Healthcare) gel-filtration column pre-equilibrated with 20 m*M* Tris–HCl pH 8.0, 100 m*M* NaCl, 2 m*M* DTT was normalized using bovine serum albumin. The individual proteins (streptavidin C1 and C2 and the C-terminal deletion mutants ΔC1 and ΔC2) were prepared separately by the methods described earlier and were injected (1–2 mg ml^−1^, 0.25 ml) at a flow rate of 0.8 ml min^−1^. The data were analyzed using the Zimm model for fitting static light-scattering data and were represented using an EASI graph with a UV peak in the *ASTRA V* software (Wyatt).

### 2.8. MALDI-TOF mass-spectrometric analysis   

Mass spectra of recombinant streptavidin C1 were measured via matrix-assisted laser desorption/ionization time-of-flight mass spectrometry (MALDI-TOF MS; eMass, Seoul, South Korea). The protein was dissolved in 40% acetonitrile at a concentration of 20 mg ml^−1^ with 0.2% trifluoroacetic acid and was purified by ZipTip C4 (Millipore). After using a dry vacuum pump and centrifugal evaporator, sinapinic acid was added to the sample as the matrix solution. The sample was applied to a MALDI plate and subjected to MALDI-TOF MS.

### 2.9. Crystallization and data collection   

Crystals of streptavidin were grown by the hanging-drop vapor-diffusion method at 20°C with a crystallization buffer consisting of 50 m*M* bis-Tris pH 6.5, 28% pentaerythritol ethoxylate (15/4 EO/OH), 150 m*M* ammonium sulfate. The protein solution (approximately 13 mg ml^−1^) consisted of 20 m*M* Tris–HCl pH 8.0, 100 m*M* sodium chloride, 2 m*M* DTT. The streptavidin protein was incubated with 3 m*M* d-biotin for 4 h at 4°C for cocrystallization and was then crystallized. The streptavidin crystals were formed at 20°C using 1 µl well solution, 0.2 *M* potassium thiocyanate, 20% PEG 3350 and 1 µl protein solution. Diffraction data were collected on BL-11C at Pohang Accelerator Light Source, Pohang, South Korea. All collected images were processed and scaled with the *HKL*-2000 package (Otwinowski & Minor, 1997 ▸).

### 2.10. Structure determination and refinement   

Streptavidin crystals in the mature form and in complex with d-biotin belonged to the tetragonal space group *P*4_2_2_1_2, with unit-cell parameters *a* = *b* = 58.442, *c* = 78.492 Å and *a* = *b* = 57.938, *c* = 74.729 Å, respectively. The asymmetric unit contained one molecule (Supplementary Fig. S5). The initial structure of streptavidin C1 was resolved by molecular replacement with *Phaser* (McCoy *et al.*, 2007 ▸) using partial models (PDB entry 2bc3; Le Trong *et al.*, 2006 ▸) as search models in the *CCP*4 suite (Winn *et al.*, 2011 ▸). Model building was performed automatically with the *AutoBuild* (Langer *et al.*, 2008 ▸) module in *Phenix* (Liebschner *et al.*, 2019 ▸) and manually with *Coot* (Emsley *et al.*, 2010 ▸). The final model refinement was performed in *REFMAC* (Murshudov *et al.*, 2011 ▸). The final models were validated using *MolProbity* (Chen *et al.*, 2010 ▸) and had *R* values of *R*_work_ = 20.3% and *R*_free_ = 22.6% for mature streptavidin C1 and *R*_work_ = 19.1% and *R*_free_ = 23.3% for the complex with biotin. The data-collection and structure-refinement statistics are summarized in Table 1 ▸. All structural figures were generated in *PyMOL* (Schrödinger). The structure-factor and coordinate files have been deposited in the Protein Data Bank with accession codes 7cq0 and 7cpz.

## 3. Results and discussion   

### 3.1. Identification of antifungal proteins from *S. cinnamonensis* strain KPP02129   

**Figure 1:**
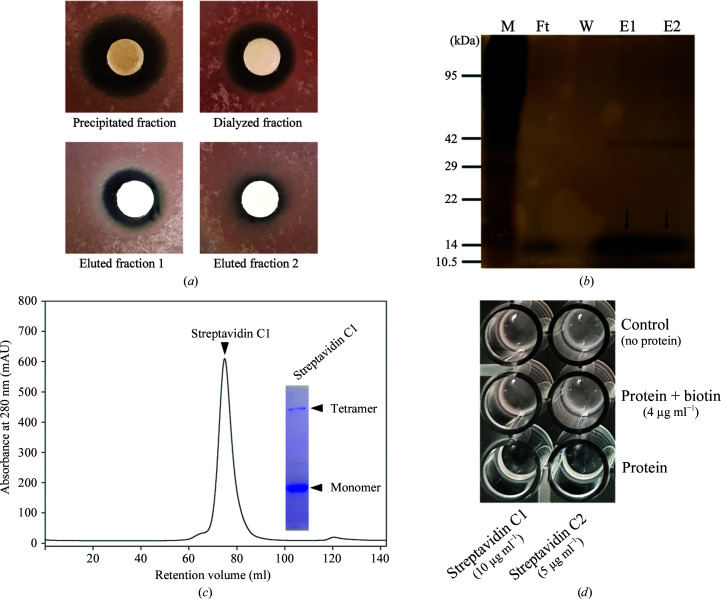
Purification of an antifungal protein from *S. cinnamonensis* strain KPP02129. (*a*) Antifungal activity of semi-purified fractions. (*b*) Silver-stained SDS–PAGE during anion-exchange chromatography. The arrow indicates the purified protein. Lane M, marker; lane Ft, flowthrough fraction; lane W, washing fraction; lane E1, eluted fraction 1; lane E2, eluted fraction 2. (*c*) Streptavidin C1 was purified via gel-filtration chromatography. Arrowheads indicate the peak and bands corresponding to streptavidin C1. (*d*) Antifungal activities of the two proteins treated with or without d-biotin against *F. oxysporum* f. sp. *cucumerinum*.

**Figure 2:**
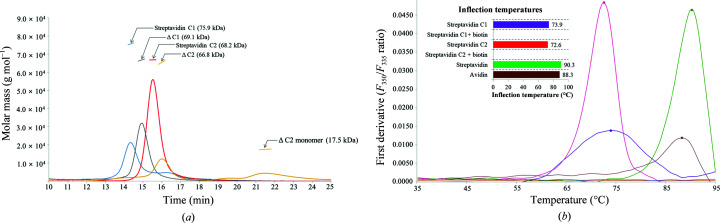
Molecular mass and thermal stability of biotin-binding proteins. (*a*) Streptavidin C1 (blue line), streptavidin C2 (red line), ΔC1 (silver line) and ΔC2 (yellow line) analyzed by SEC-MALS. All samples were measured at a concentration of 1–2 mg ml^−1^. The horizontal line represents the measured molar mass. Experimental (MALS) molar-mass values are indicated as arrows. (*b*) Thermal unfolding profile analysis of streptavidin C1 (violet), streptavidin C2 (red), streptavidin (green) and avidin (brown) with or without biotin from differential scanning fluorimetry.

Firstly, for the identification of potent antifungal proteins against *F. oxysporum* f. sp. *cucumerinum* (FOC), we used the culture broth of *S. cinnamonensis* strain KPP02129 (deposited in GenBank as accession No. MT890000) cultured in modified TSB. To identify its active ingredients, we performed conventional ammonium sulfate precipitation and dialysis as described in Section 2. Thereafter, two active fractions, eluted fraction 1 (ET1; 20 m*M* Tris–HCl pH 8.0, 100 m*M* NaCl, 2 m*M* DTT) and eluted fraction 2 (ET2; 20 m*M* Tris–HCl pH 8.0, 150 m*M* NaCl, 2 m*M* DTT), exhibited antifungal activity against FOC [Figs. 1 ▸(*a*) and 1 ▸(*b*)]. The bands were excised from a Coomassie-stained gel and were treated with trypsin for protein identification (eMass, Seoul, South Korea). Eventually, via protein identity analysis, the antifungal proteins were identified to be avidin-family proteins. To characterize these new avidin-like proteins (named strept­avidin C1 and C2), the full-length open reading frame (ORF) of streptavidin C1 (Met1–Glu191) from gDNA of *S. cinnamo­nensis* was cloned into the expression vector pET-21a. The recombinant protein was expressed in a soluble form in *E. coli* BL21 (DE3) cells. The final purified protein was concentrated and injected onto a Superdex 200 26/60 gel-filtration column [Fig. 1 ▸(*c*)]. After purification, streptavidins C1 and C2 were subjected to size-exclusion chromatography using multi-angle light scattering (SEC-MALS) to evaluate the exact molar mass and root-mean-square (r.m.s.) radius *R*_g_ in solution. The molecular masses determined by SEC-MALS are approximately 76 kDa and approximately 68 kDa, respectively, which correspond to a substantially tetrameric state [Fig. 2 ▸(*a*)]. Moreover, monomer and tetramer bands were observed for streptavidin C1 via SDS–PAGE when the excess sample was treated at 95°C using reducing agents [Fig. 1 ▸(*c*)]. SDS–PAGE analysis of purified streptavidin C1 revealed molecular masss of approximately 18 kDa for the monomer and approximately 76 kDa for the tetramer, which were consistent with those observed by SEC-MALS. We performed N-terminal sequencing to identify the appropriate mature sequences of streptavidin C1. The result clearly indicated that the N-terminal sequence of the processed protein is –^34^SADPR^38^– (Supplementary Fig. S1). Mass-spectrometric analysis by MALDI-TOF MS of recombinant streptavidin C1 revealed a large peak at *m*/*z* = 18 667.791, corresponding to the exact molecular weight of the monomeric protein (Supplementary Fig. S2). The purified antifungal proteins were tested against FOC using a 96-well plate assay. The germination of *Fusarium* spores was inhibited at a concentration of approximately 10 µg ml^−1^ streptavidin C1, whereas streptavidin C2 exhibited the strongest antifungal activity at a concentration of approximately 5 µg ml^−1^. Furthermore, these antifungal activities disappeared on treatment with 4 µg ml^−1^ d-biotin [Fig. 1 ▸(*d*)]. These results indicate that streptavidin C1 and C2 as evaluated in the present study are biotin-binding proteins similar to other members of the (strept)avidin family.

### 3.2. Overall structure of streptavidin C1   

**Figure 3:**
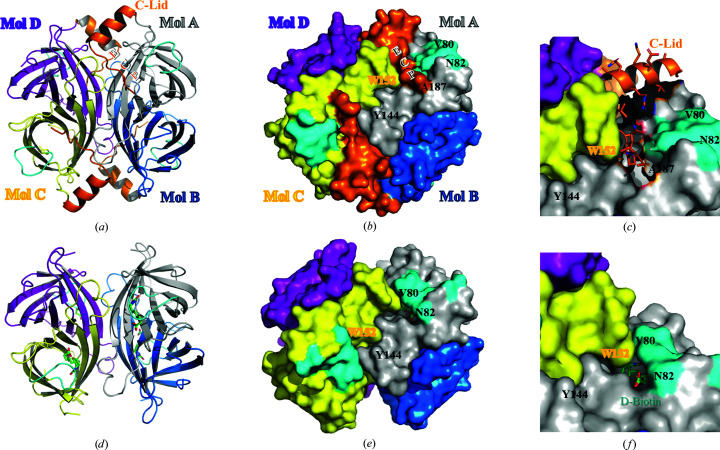
Overall structure of streptavidin C1. (*a*) Overall tetrameric structure of streptavidin C1. The C-terminal region named C-Lid and the flexible loop region (Loop^3–4^) are represented as orange and cyan cartoon models, respectively. The monomers are labeled Mol A, Mol B, Mol C and Mol D and are colored gray, marine, yellow and purple, respectively. (*c*) Surface representation of the streptavidin C1 tetramer. Mol A and Mol B (as well as Mol C and Mol D) closely interact to form a stable dimer, and two dimers assemble into a tetramer. The color code is the same as in (*a*). (*c*) Enlarged view of C-Lid and ECP, which interact with various hydrophobic residues such as Leu58, Val80, Trp140, Phe156 (Mol A) and Trp152 (located on Loop^7–8^ of the neighboring Mol C). (*d*) Overall tetrameric structure of streptavidin C1 complexed with biotin. Biotin is represented as a ball-and-stick model in green. (*e*) Surface representation of tetrameric streptavidin C1 complexed with biotin. (*f*) Enlarged view of the biotin-binding site of streptavidin C1.

**Figure 4:**
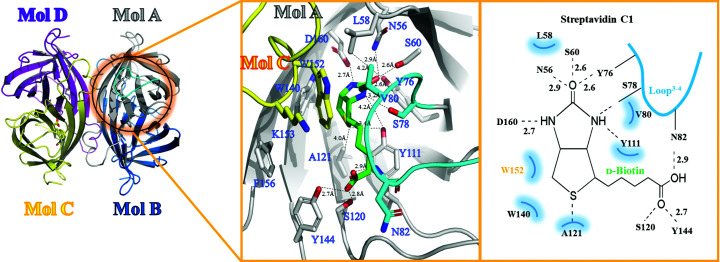
Enlarged view of the streptavidin C1 structure complexed with biotin. The hydrogen-bonding network with biotin is formed by Asn56, Ser60, Tyr76, Ser78 and Asp160, and hydrophobic interactions occur between biotin and strictly conserved residues (Leu58 in Loop^1–2^, Val80 in Loop^3–4^, Tyr111 in Loop^5–6^, Trp140 in β7 and Leu142 in β7). Residues in the flexible Loop^3–4^ (–^80^VGN^82^–) play a pivotal role in the binding of biotin, which is mainly formed with Trp152 (Mol C) in a closed conformation.

**Figure 5:**
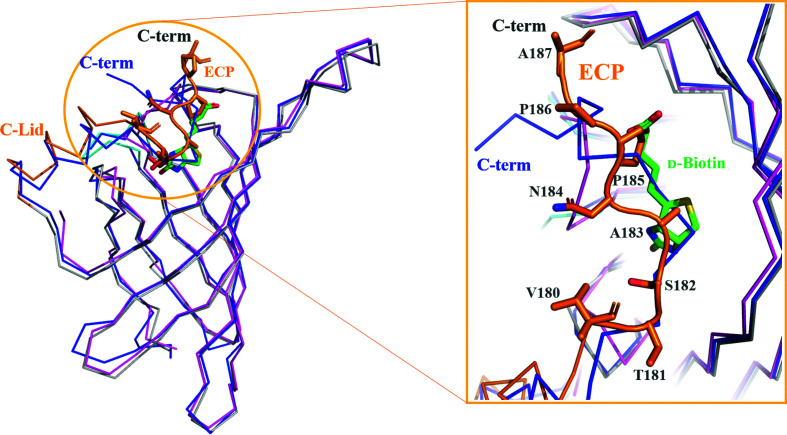
Structural comparison with other (strept)avidin family members. Left: superimposition of the overall structures of streptavidin C1, streptavidin (PDB entry 2bc3) and avidin (PDB entry 5irw). Streptavidin C1, streptavidin and avidin are represented by gray, blue and magenta ribbon models, respectively. The extension C-terminal peptide (ECP) is represented as an orange cartoon model and d-biotin is represented by a green stick model. Right: enlarged view of the ECP (amino acids 180–191), which stretches into the biotin-binding site of the same monomer. When superimposed on streptavidin, the ECP region, which is the binding site for d-biotin, reveals a similar binding site in streptavidin C1 and streptavidin. Most of the amino-acid sequence of the ECP differs from that of streptavidin, apart from the –^184^NP^185^– sequence.

To gain structural insight into the biotin-binding site and the long C-terminal region of mature streptavidin C1, the recombinant intact mature form was expressed and purified for crystallization. We determined crystal structures of strept­avidin C1 (191 amino acids) in the mature form and as a complex with biotin at 2.1 and 2.5 Å resolution, respectively (Fig. 3 ▸). The asymmetric unit comprised one molecule and clearly indicated a tetrameric molecule with *D*_2_ symmetry in the crystal [Fig. 3 ▸(*a*)]. Each monomer contains eight antiparallel β-strands that form a classical β-barrel structure (Hendrickson *et al.*, 1989 ▸; Weber *et al.*, 1989 ▸). Mol A and Mol B [as well as Mol C and Mol D in Fig. 3 ▸(*b*)] closely interact to form a stable dimer, and two dimers assemble into a tetramer via hydrophobic interactions involving Leu58, Val80, Trp140 (Mol A) and Trp152 (located on Loop^7–8^ of the neighboring Mol C; Fig. 4 ▸, Supplementary Fig. S4). Similar to other streptavidin family members, four sets of interactions are present in the biotin-binding sites. Firstly, a hydrogen-bonding network with biotin is formed by Asn56, Ser60, Tyr76, Ser78 and Asp160. The second set of interactions comprises hydrophobic interactions between biotin and strictly conserved residues (Leu58 in Loop^1–2^, Val80 in Loop^3–4^, Tyr111 in Loop^5–6^, Trp140 in β7 and Leu142 in β7). Thirdly, residues in the flexible Loop^3–4^ (–^80^VGN^82^–) play a pivotal role in the binding of the biotin lid, which is mainly formed with Trp152 (Mol C) in a closed conformation (Fig. 4 ▸). Fourthly, the pocket in the tetrameric structure is formed with hydrophobic contributions from the other protomers in the subunit (Mol C and Mol D) [Figs. 3 ▸(*e*) and 3(*f*)]. Moreover, the long C-terminal region comprises a short α-helix (C-Lid; amino acids 169–179) and an extension C-terminal peptide (ECP; amino acids 180–191) which stretches into the biotin-binding sites of the same monomer. To date, only one mature structure of the streptavidin family with an extension C-terminal peptide has been reported (Le Trong *et al.*, 2006 ▸). We did not find electron density for the ECP and C-Lid regions when biotin was bound to the protein, which indicates that these regions are flexible but do not affect the binding ability of biotin. In the mature crystal structure, the C-Lid and ECP mainly interact with His119 in Loop^5–6^ of Mol B′ (where ′ indicates the symmetric neighboring molecule) and Arg135 of Mol D′ (Supplementary Fig. S6); however, the binding site for the ECP is almost identical to that of d-biotin (Fig. 5 ▸). Therefore, the ECP sequence (–^180^VTSANPPAS^188^–) is a newly defined biotin-binding site which reduces the binding ability of (strept)avidin family proteins.

### 3.3. Thermal stability of streptavidins C1 and C2   

The thermal stabilities of recombinant streptavidins C1 and C2 were studied under diverse temperature conditions using an SDS–PAGE system and Tycho NT.6 (NanoTemper Technologies), and were further compared with those of other biotin-binding proteins. Firstly, recombinant streptavidin C1 heat-treated at 25–80°C revealed tetrameric and monomeric forms on SDS–PAGE, whereas recombinant streptavidin C2 showed both forms at 60–80°C and only the tetrameric form at 25–50°C [Supplementary Figs. S3(*a*) and S3(*b*)]. The proteins only existed in a monomeric form at 90–100°C. The transition temperatures (*T*_r_) of streptavidins C1 and C2, in which half of the protein is tetrameric and other half is monomeric, in the absence of biotin, were 60 and 70°C, respectively. The *T*_r_ value of streptavidin C1 is lower than that of streptavidin C2 and the reported values for streptavidin, bradavidin and tamavidin (Bayer *et al.*, 1996 ▸; Nordlund, Hytönen, Laitinen *et al.*, 2005 ▸; Takakura *et al.*, 2009 ▸). Moreover, streptavidins C1 and C2 with d-biotin exhibited thermal stability even at 100°C and mostly remained in the tetrameric form [Supplementary Figs. S3(*c*) and S3(*d*)]. Furthermore, the thermal stabilities of the proteins in the presence of d-biotin and β-mercaptoethanol were extremely high [Supplementary Figs. S3(*e*) and S3(*f*)]. The disulfide bridge between Cys4 and Cys83 in Loop^5–6^ is a common distinctive feature in all dimeric avidins analyzed to date (Meir *et al.*, 2012 ▸) and thus contributes to their conformational stability. In our proteins no cysteine residues are present, and Pro48 (corresponding to Cys4 of avidin) and Tyr128 (corresponding to Cys83 of avidin) are located at similar positions to those in other members of the avidin family (Supplementary Fig. S4). Therefore, the thermal stabilities of both streptavidins C1 and C2 are not affected by the presence of β-mercaptoethanol, as a strong hydrophobic and hydrogen-bonding interaction is formed instead of a disulfide bridge in the structure (Supplementary Figs. S4 and S5). Secondly, a thermal shift assay was performed using Tycho NT.6 for both mature streptavidins C1 and C2 in the apo and biotin-complexed forms [Fig. 2 ▸(*b*)]. The fluorescence signals are plotted as a ratio (350/330 nm) and are used to calculate the midpoint unfolding inflection temperature (*T*_i_). Streptavidins C1 and C2 demonstrate high thermostability, although their *T*_i_ values were lower than those normally observed for proteins of the (strept)avidin family at 73.9 and 72.6°C, respectively, for the mature form and above 95°C for the biotin complexes. Streptavidin and avidin were evaluated, and their *T*_i_ values were higher than those of streptavidins C1 and C2 (90.3 and 88.3°C for streptavidin and avidin, respectively).

### 3.4. Structural comparison with other (strept)avidin family members   

The sequence identities between streptavidin C1 (amino acids 1–191) and streptavidin (PDB entry 2bc3; Le Trong *et al.*, 2006 ▸) and avidin (PDB entry 5irw; Strzelczyk *et al.*, 2018 ▸) are approximately 65.4% and 32.4%, respectively (Fig. 5 ▸, Supplementary Figs. S4 and S5). Both streptavidin C1 and avidin have structural similarity to streptavidin, with root-mean-square (r.m.s.) deviations of 1.10 and 1.33 Å, respectively, when 140 C^α^ atoms of streptavidin were aligned in *Coot* (Emsley *et al.*, 2010; Fig. 5 ▸, Supplementary Fig. S5). With regard to the differences in the dimeric avidin structure, as described in Section 3.3, no cysteine residues are present in streptavidin C1. Tyr128 interacts with several residues at the N-terminus in the crystal structure (Supplementary Fig. S5). Tyr111 in Loop^5–6^ is a specific novel residue that interacts with d-biotin, and in most proteins of the (strept)avidin family this position is occupied by a tryptophan residue, except for tamavidin, which has a phenylalanine at this position. One of the important structural differences between streptavidin C1 and streptavidin is the ECP region, which is a binding site for d-biotin when superimposed on streptavidin (Fig. 5 ▸). Notably, the ECP is located near the d-biotin-binding site. This self-binding of the ECP from streptavidin C1 is considerably further from the d-biotin-binding site than that in streptavidin. Most of the amino-acid sequence of the ECP differs from those of streptavidin (Fig. 5 ▸). Only two amino acids (–NP–) of the ECP are similar to those in streptavidin. Considering the sequence and structural similarities, we suggest that this ECP sequence (–^180^VTSANPPAS^188^–) is a newly defined biotin-binding site which reduces the binding ability of the (strept)avidin family proteins, with the –NP– sequence possibly acting as the key sequence in this binding site. To effectively reduce the biotin-binding ability, we will further design a peptide based on our structural analysis of streptavidin C1.

### 3.5. Affinity of streptavidins for binding to biotin   

**Figure 6:**
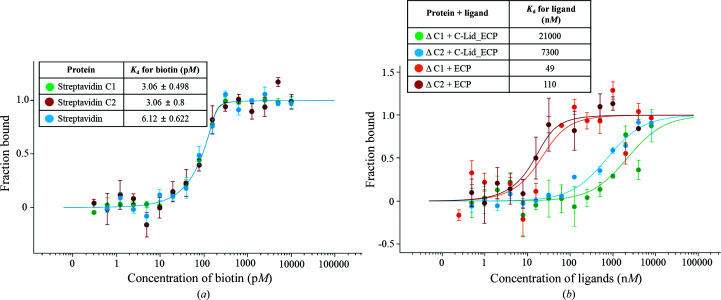
Direct interactions between fluorescently labeled streptavidins and biotin or labeled C-terminal deletion mutants and ligands measured by microscale thermophoresis (MST). (*a*) Dose–response curves of streptavidin C1 (green), streptavidin C2 (red) or streptavidin (blue) and the biotin molecule. (*b*) Dose–response curves of ΔC1 + C-Lid_ECP (green), ΔC2 + C-Lid_ECP (blue), ΔC1 + ECP (orange) and ΔC2 + ECP (red). The amino-acid sequences of C-Lid_ECP and ECP were designed as –^169^SAADVEKARQLGVTSANPPASDGE^191^– and –^178^LGVTSANPPASDGE^191^–, respectively.

The interaction between streptavidin C1 or two deletion mutants (ΔC1, amino acids 1–161, and ΔC2, amino acids 1–158) and d-biotin was measured using microscale thermophoresis (MST), a novel method for the quantitative analysis of protein interactions in free solution. Recombinant strept­avidins C1 and C2 and streptavidin proteins (target concentration of 200 p*M*) were labeled with fluorescent dye and incubated with increasing concentrations of biotin (0.3052–10 000 p*M*) [Fig. 6 ▸(*a*)]. The *K*_d_ of biotin binding to recombinant streptavidin C1 was about 3.06 ± 0.498 p*M*. The apparent *K*_d_ values of streptavidin C2 and streptavidin for d-biotin were 3.06 ± 0.8 and 6.12 ± 0.622 p*M*, respectively [Fig. 6 ▸(*a*)]. These results suggest that streptavidins C1 and C2 have an extremely high affinity for d-biotin, similar to that of streptavidin. In this study, we constructed two deletion mutants, ΔC1 and ΔC2, in order to identify the binding affinities of C-Lid_ECP and ECP, respectively [Fig. 6 ▸(*b*)]. The results reveal that the ECP sequence has a higher binding affinity than the C-Lid_ECP sequence for ΔC1 and ΔC2. The *K*_d_ values of C-Lid_ECP and ECP for ΔC1 are approximately 21 000 and 49 n*M*, respectively. The *K*_d_ values of ΔC2 are about 7300 n*M* and 110 n*M*, respectively [Fig. 6 ▸(*b*)]. We suggest that the ECP sequence contributes greatly to the binding to streptavidin C1 or C2 compared with C-Lid_ECP. Therefore, the ECP sequence (–^180^VTSANPPAS^188^–) is a newly defined biotin-binding site which reduces the binding ability of (strept)avidin family members. The newly identified streptavidin C1 will help to develop an engineered tetrameric streptavidin with reduced biotin-binding capability and of other desirable biomaterial tools.

## 4. Conclusion   

This study showed that streptavidins C1 and C2, which are new antifungal proteins from *S. cinnamonensis* strain KPP02129, could inhibit mycelial growth of *F. oxysporum* f. sp *cucumerinum*. We assessed the crystal structures of strept­avidin C1 in the mature form and as a complex with biotin at 2.1 and 2.5 Å resolution, respectively. We also measured the thermal stability and binding affinity of streptavidins C1 and C2 by MST. Interestingly, we found that the ECP sequence (–^180^VTSANPPAS^188^–) is a newly defined biotin-binding site which reduces the binding ability of (strept)avidin family members. The novel streptavidin C1 could help in the develop­ment of an engineered tetrameric streptavidin with a reduced biotin-binding capacity as well as other biomaterial tools.

## Supplementary Material

PDB reference: streptavidin C1, mature form, 7cq0

PDB reference: complex with d-biotin, 7cpz

Supplementary Tables and Figures. DOI: 10.1107/S2052252520015675/lz5046sup1.pdf

## Figures and Tables

**Table 1:** Data-collection and refinement statistics Values in parentheses are for the highest resolution shell.

	Streptavidin C1 (PDB entry 7cq0)	Streptavidin C1, D-biotin complex (PDB entry 7cpz)
Data collection		
Space group	*P*4_2_2_1_2	*P*4_2_2_1_2
*a*, *b*, *c* (Å)	58.442, 58.442, 78.492	57.938, 57.938, 74.729
α, β, γ (°)	90, 90, 90	90, 90, 90
Resolution (Å)	41.54–2.10 (2.14–2.10)	41.02–2.50 (2.54–2.50)
Completeness (%)	99.4 (87.7)	99.7 (100)
Mulitplicity	22.2 (12.4)	24.7 (26.1)
〈*I*/σ(*I*)〉	6.3 (0.9)	7.5 (1.9)
*R*_merge_ (%)†	6.6 (49.1)	6.7 (18.9)
Refinement statistics		
Resolution (Å)	46.88–2.03	41.02–2.50
*R*_work_/*R*_free_ (%)	20.3/22.6	19.1/23.3
*B* factors (Å^2^)		
Average	31.34	28.57
Protein	30.93	27.68
Ligands		25.39
Solvent	35.81	36.69
R.m.s. deviations		
Bond lengths (Å)	0.002	0.002
Bond angles (°)	0.536	0.587
Ramachandran plot (%)		
Favored	97.10	98.31
Allowed	2.90	1.69
Outliers	0.00	0.00

† *R*_merge_ = 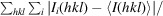 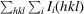.
