# Supplementary material for: Insights into the structure of mature streptavidin C1 from Streptomyces cinnamonensis reveal the self-binding of the extension C-terminal peptide to biotin-binding sites
Source: IUCrJ. 2021 Jan 11;8(Pt 2):168–77. doi: 10.1107/S2052252520015675 (PMC7924230; doi:10.1107/S2052252520015675)
Supplement: Supplementary file 1 [file m-08-00168-sup1.pdf]

IUCrJ

Volume 8 (2021)

Supporting information for article:

Insights into the structure of mature streptavidin C1 from *Streptomyces cinnamonensis* reveal the self-binding of the extension C-terminal peptide to biotin-binding sites

Byeong Jun Jeon, Sulhee Kim, Min-Seok Kim, Ji-Ho Lee, Beom Seok Kim and Kwang Yeon Hwang

Table S1 Primers used in this study. Restriction sites are shown in italic type.

Primer	Sequence (5′- to ′3)
C1F-NdeI	*CATATG*GTGACGCGTGTACGCCAA
C1R-XhoI	*CTCGAG*CTCCCCGTCGGAGGC
C2F-NdeI	*CATATG*GTGTCGCACATGCGCAAGATCG
C2R-XhoI	*CTCGAG*CTGCTGGACGGCGTCGAGCG
ΔC1R-XhoI	*CTCGAG*GGGCTTGACCCGGGTGAA
ΔC2R-XhoI	*CTCGAG*CGGCTTCACCTTGGTGAA

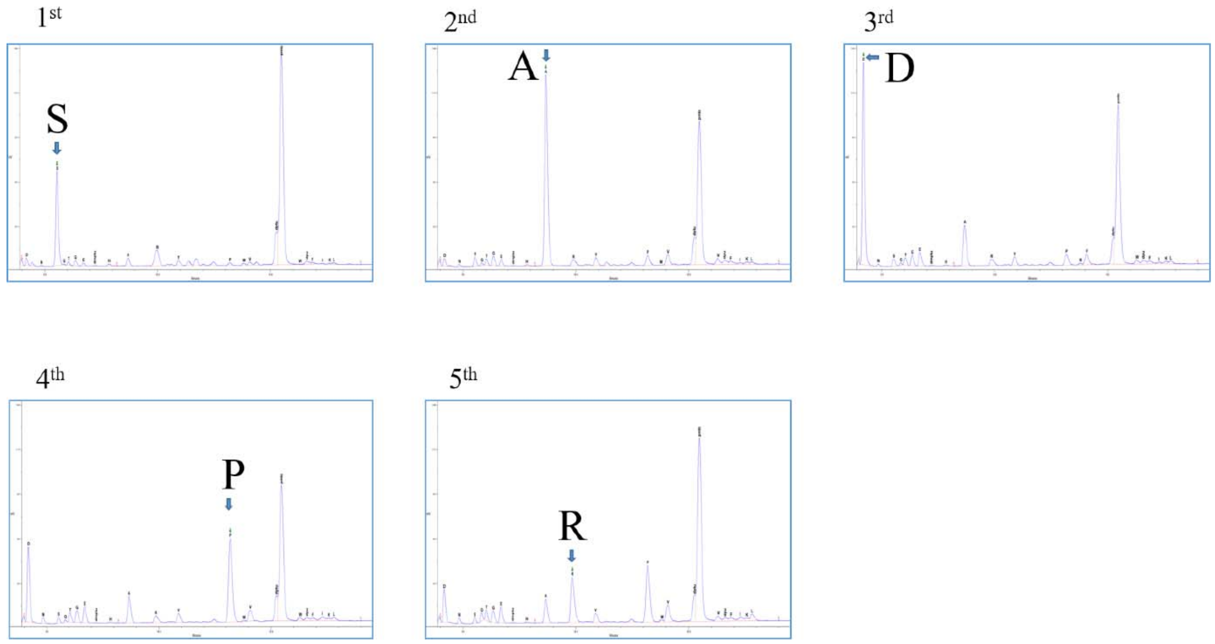

Figure S1 Determination of the N-terminal sequence of recombinant streptavidin C1 using the standard Edman degradation method. The first five amino acids in the N-terminal region of streptavidin C1 were evaluated. Diphenylthiourea is the byproduct of the Edman degradation reaction.

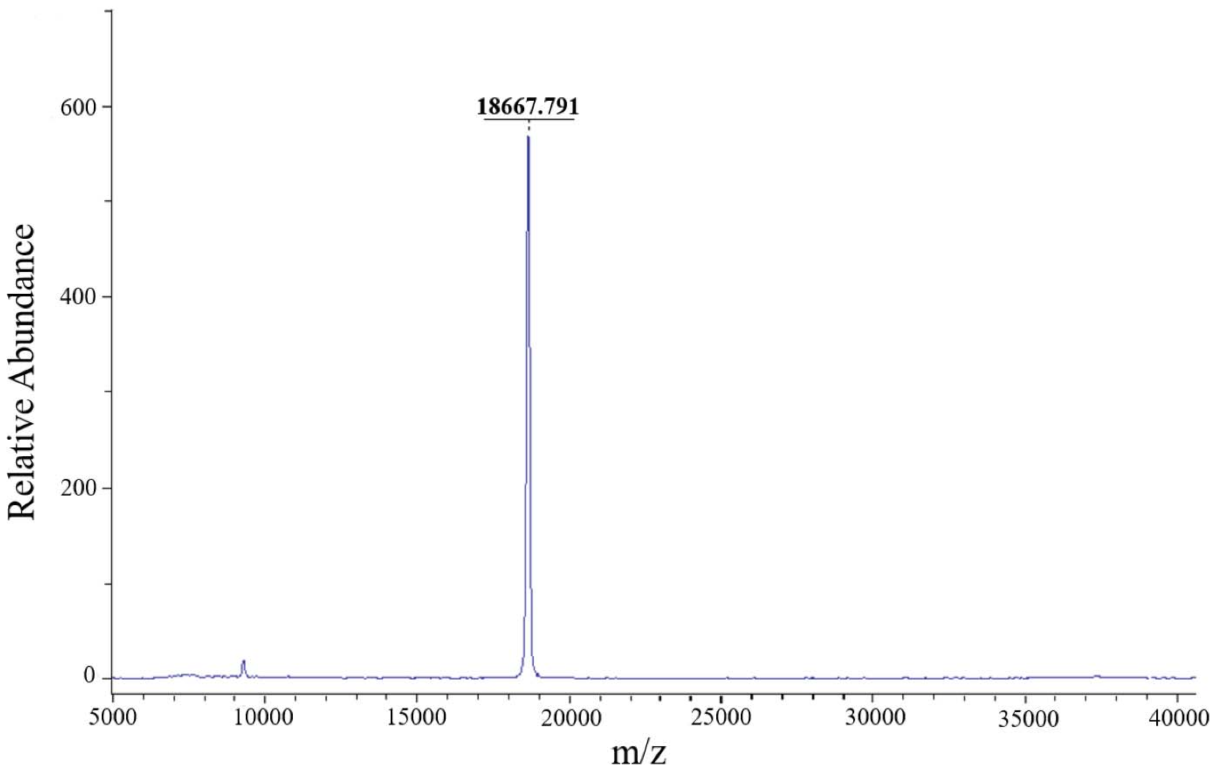

Figure S2 MALDI-TOF mass spectrum of recombinant streptavidin C1. The measurement was performed via MALDI-TOF mass spectrometer using bovine serum albumin as an external standard calibration and sinapinic acid as a matrix.

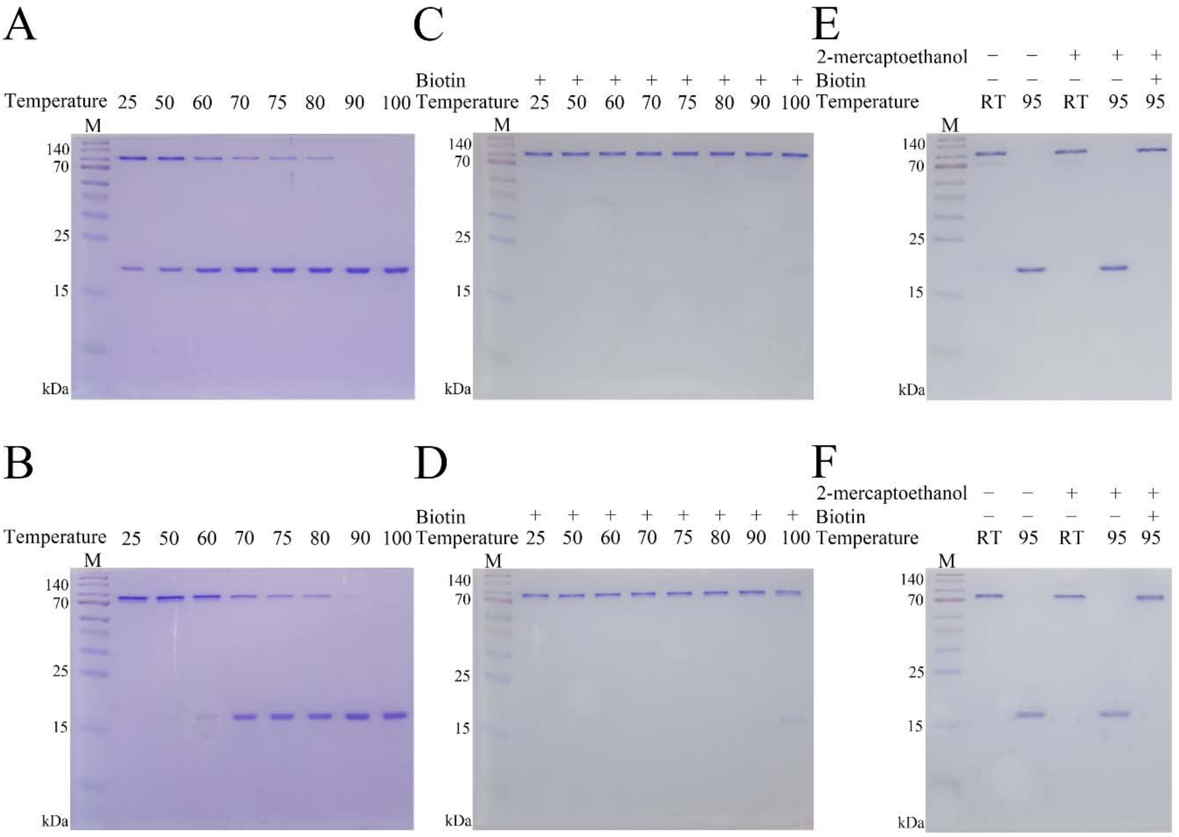

Figure S3 Thermal stability of streptavidin C1 and streptavidin C2 expressed in *Escherischia coli*. Transition temperatures (Tr) of (*A*) streptavidin C1 and (*B*) streptavidin C2 at various temperatures without D-biotin. Transition temperatures (Tr) of (*C*) streptavidin C1 and (*D*) streptavidin C2 at various temperatures with D-biotin. (*E*) Streptavidin C1 and (*F*) streptavidin C2 were incubated with or without β-mercaptoethanol in 1× SDS sample buffer at room temperature (RT) or at 95 °C with or without biotin.

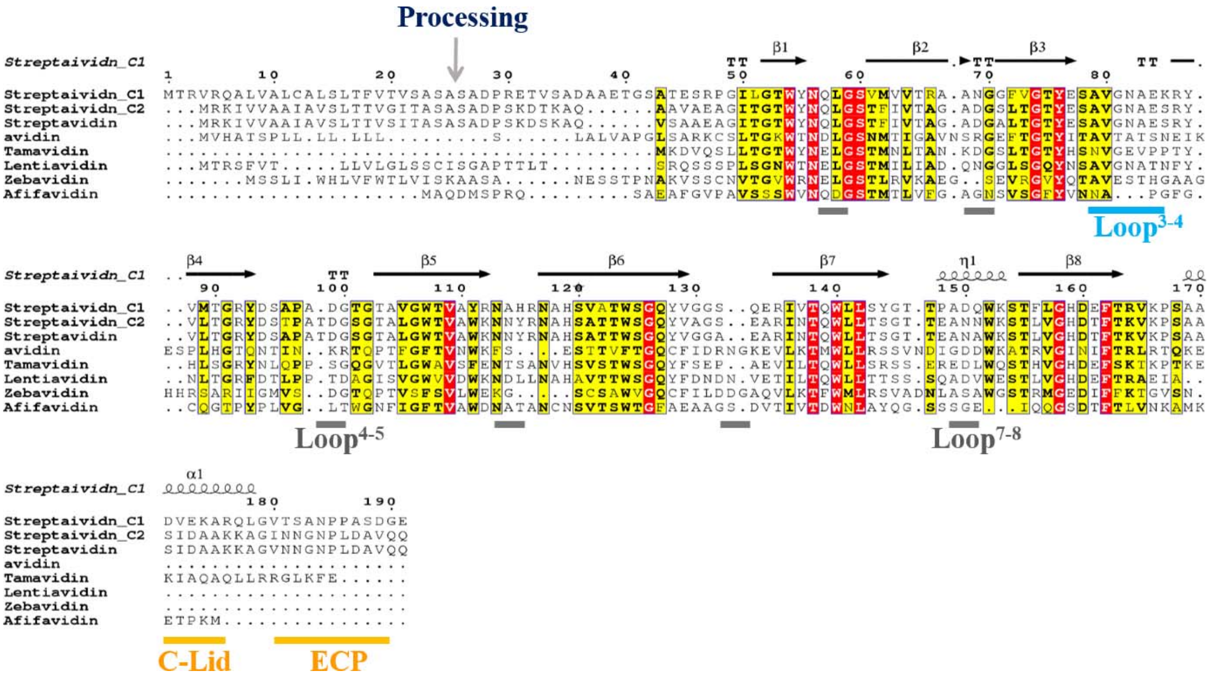

Figure S4 Multiple sequence alignment of streptavidin C1 (Streptomyces sp. H036, Uniprot entry: A0A0M8UVL7), streptavidin C2 (Streptomyces sp. H036, Uniprot entry: A0A0N0SUI3), streptavidin (Streptomyces avidinii, Uniprot entry: P22629), avidin (Gallus gallus, Uniprot entry: P02701), tamavidin (Pleurotus cornucopiae, Uniprot entry: B9A0T6) , lentiavidin (Lentinula edodes, Uniprot entry: A0A0P0YK50), zebavidin (Danio rerio (Zebrafish), Uniprot entry: E7F650), and afifavidin (Afilfella pfennigii, Uniprot entry: A0A493R6X0). Complete identity is illustrated in red boxes and difference in similarity in yellow boxes.

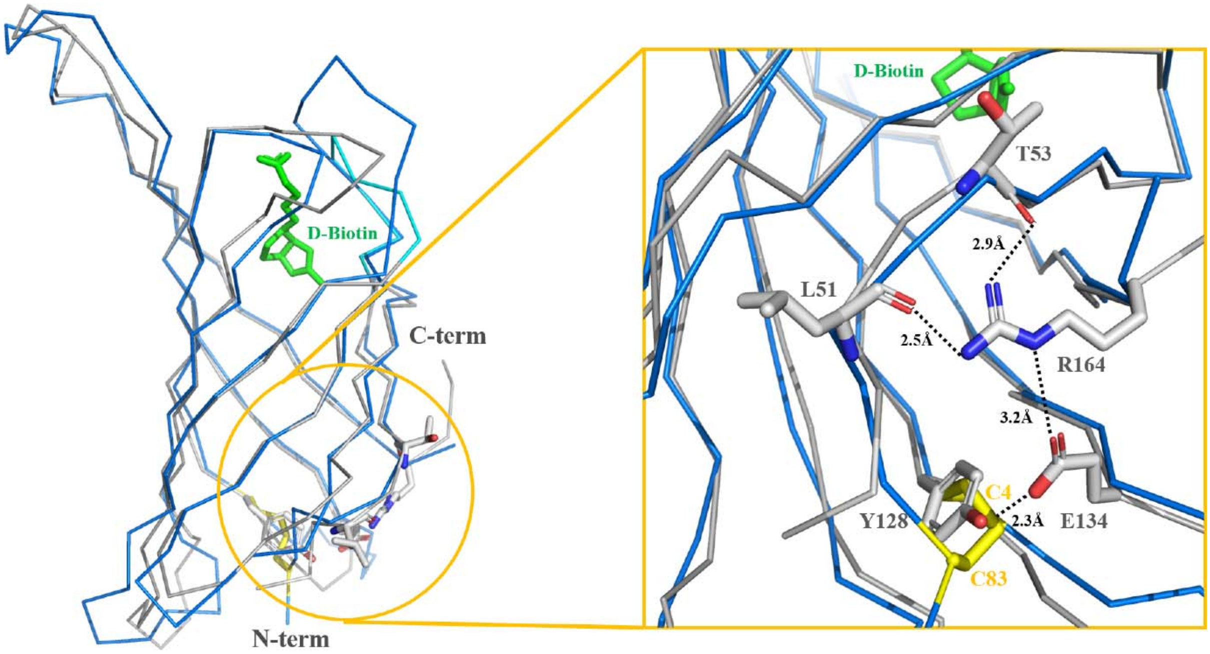

Figure S5 The structural comparison of streptavidin C1 and dimeric avidin structure (PDB entry: 5irw). Streptavidin C1 is different from the dimeric avidin structure as it lacks a cysteine residue. Y128, instead of the cysteine residue, interacts with several residues (E134, R164, and L51) in the crystal structure.

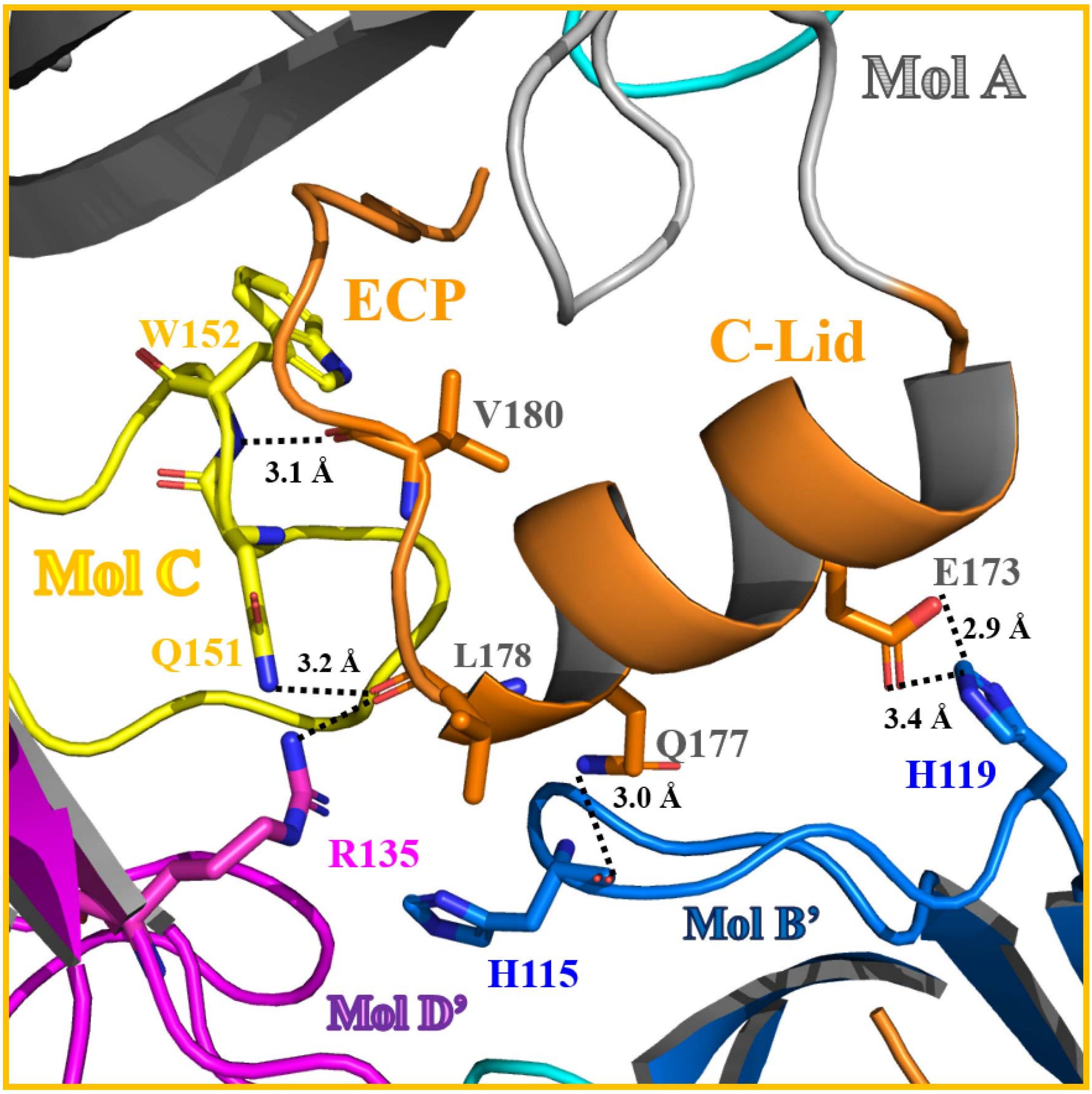

Figure S6 Zoomed view of neighbouring residues of C-Lid and ECP with crystallographic symmetry protomers (Mol B' and Mol D').
